# The Influence of Abrasive and Acidic Aggressions on the Surface Condition of Flowable Composite Resin

**DOI:** 10.3390/ma15031000

**Published:** 2022-01-27

**Authors:** Ionuț Tărăboanță, Simona Stoleriu, Silviu Gurlui, Irina Nica, Andra Claudia Tărăboanță-Gamen, Alexandru Iovan, Sorin Andrian

**Affiliations:** 1 Faculty of Dental Medicine, Grigore T. Popa University of Medicine and Pharmacy, 16 Universitatii Str., 700115 Iaşi, Romania; ionut-taraboanta@umfiasi.ro (I.T.); irina.nica@umfiasi.ro (I.N.); alexandru.iovan@umfiasi.ro (A.I.); sorin.andrian@umfiasi.ro (S.A.); 2 Faculty of Physics, Atmosphere, Optics, Spectroscopy and Lasers Laboratory (LOA-SL), Alexandru Ioan Cuza University, 11 Carol I Str., 700506 Iaşi, Romania; sgurlui@uaic.ro

**Keywords:** flowable composite resin, hydrochloric acid, surface roughness, toothbrush

## Abstract

The aim of this in vitro study was to evaluate the effect of hydrochloric acid associated with the abrasive effect of toothbrushing on the surface condition of three flowable composite resins used for direct restoration. Seventy samples of each composite resin: Grandio Flow (VOCO, Germany)—group A, Filtek Ultimate Flow (3M-ESPE, MN, USA)—group B, G-aenial Flo X (GC Europe)—group C were prepared, submersed in hydrochloric acid 30% for 60 min and then submitted to simulated toothbrushing procedure using 5000 cycles with toothbrushes with medium and hard bristles, immediately after the chemical attack, after 30 min or without any chemical attack. The sample’s surface roughness was analyzed using a noncontact profilometer (Dektak XT, Bruker, USA). ANOVA and post hoc Bonferroni tests, with a *p* < 0.05, were used to analyze the values. Hydrochloric acid action for 60 min and six months of toothbrushing using toothbrushes having medium hardness or firm bristles affects the surface roughness of tested flowable composite resins. Toothbrushing with firm bristles immediately after acidic challenge determines increased surface roughness for two of the three flowable composite resins (Grandio Flow and Filtek Ultimate Flow). Toothbrushing with medium or firm bristles thirty minutes after the acidic aggression determine no effect on surface condition of flowable composite resins.

## 1. Introduction

Direct dental restorations should meet two goals: aesthetics and functionality [1]. Therefore, resin-based materials have become the most preferred materials for direct anterior and posterior restorations. Since their introduction on the market until now, these materials have undergone many changes regarding the organic matrix or the type of polymerization, but the changes have mainly focused on their filling technology [2]. The reduction in the volume of inorganic phase of composite resins has led to the appearance of low viscosity or flowable composite resins. Although originally intended as restorative materials for V Black Class cavities, flowable composite resins have been accepted for a variety of applications due to their simple maneuverability and their fluidity, which allow the material to reach difficult cavity areas regarding the access or low modulus of elasticity for a uniform distribution and attenuation of occlusal forces [2,3].

The durability of composite resin restorations in oral cavity depends on different factors such as marginal adaptation or surface wear, which can be influenced by the conditions provided by the oral environment such as the erosion process, for instance [1]. Oral acidity is produced by extrinsic or intrinsic acids such as gastric acid in gastroesophageal reflux disease or acids resulting from the degradation of polysaccharides into acids [1,4]. Therefore, the chemical characteristics of the oral environment can negatively influence the restorative materials condition as a result of dissolution and disintegration process [1,5].

Wear was defined by Jones et al. as a progressive loss of substance from the surface of a body as a result of mechanical action [6]. Just like erosion, wear can cause changes in the surface condition of materials or can compromise marginal adaptation [3]. Although toothbrushing is the most effective method to control bacterial biofilm, its action can cause degradation of dental tissues and restorative materials [3,5]. Previous studies have shown that toothbrushing increases the surface roughness of composite resins resulting in material degradation and impairs clinical performance of the restorations. Composite resins are composed of inorganic fillers and an organic matrix bound together by a coupling agent, and the stress created by the brushing procedure can weaken this bond and degrade the polymer matrix, exposing the filler particles [7]. Along with the percentage of volume and weight of inorganic particles, the wear degree of a composite resin depends on the type of polymeric matrix and the degree of its polymerization [7,8].

A rough surface can negatively influence the optical properties of the material, causing changes in the degree of light absorption and reflection [2], can determine bacterial biofilm accumulation and can increase the risk for secondary caries onset. Bollen et al. concluded that the critical value of surface roughness for bacterial adhesion is 0.2 μm [9]. A smooth surface of the restoration maintains the aesthetic properties, reduces the accumulation of bacterial biofilm and is durable [10]. For these reasons, it is important to evaluate the effect of acid challenging in association with mechanical process of toothbrushing, as this is the most common habit of oral hygiene [5].

The aim of this study was to evaluate the surface roughness of three flowable composite resins after the acid attack of hydrochloric acid followed by brushing immediately after or 30 min after the chemical attack using toothbrushes with different hardness of the bristles.

## 2. Materials and Methods

**Figure 1:**
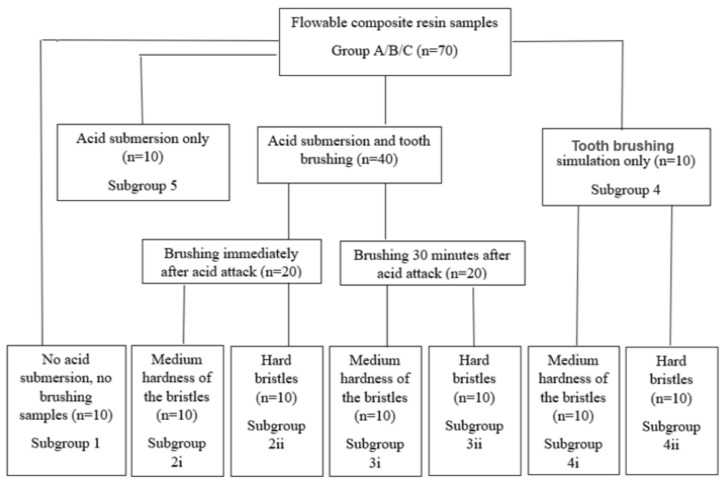
Study design.

The study design is presented in Figure 1. The details regarding the materials used in the study are shown in Table 1.

### 2.1. Sample Preparation

Seventy cylindrical samples of each material were prepared and included in three groups: group A (Grandio Flow, VOCO GmbH, Cuxhaven, Germany), group B (Filtek Ultimate Flow, 3M-ESPE, St. Paul, MN, USA) and group C (G-aenial Flo X, GC Europe, Alcobendas, Spain). The distribution of the samples and the study stages are detailed in Table 2. The samples having 2 mm height and 6 mm diameter were obtained using acrylic molds. The mold was placed on a glass plate, and then it was filled with the restoration material and covered with another glass plate. A transparent matrix was placed between the material and the glass plates in order to create smooth surfaces. A constant pressure was applied to the glass plate for 30 s, using a weight of 500 g, in order to remove the excess material and the air voids. Then the composite resin was light-cured for 40 s through the thickness of the glass plate using a LED light-curing lamp (Woodpecker LED.E, Guilin, Guangxi, China) with a light intensity of 1000 mW/cm^2^ and a wavelength range from 420 to 480 nm.

### 2.2. Finishing and Polishing Procedure

After removal from the acrylic molds, all the samples were finished using a Sof-Lex finishing and polishing system (Batch No. NC11342, 3M ESPE, MN, USA). This system is composed of two disposable wheels made of a thermoplastic elastomer impregnated with aluminum oxide particles. The beige spiral wheel is recommended for finishing, smoothing and removing the scratches produced during the restoration stages, while the white wheel is recommended for final polishing. During the finishing and polishing stage, each spiral wheel was used only once for each sample, and the procedure was performed for 1 min per sample (30 s for each wheel). The wheels were activated by a contra-angle handpiece at a speed of 20,000 revolutions per minute, according to the recommendations offered by the manufacturer.

After that, forty samples from each group were subjected to submersion in hydrochloric acid. Ten samples from groups A, B and C were maintained as they resulted after finishing and polishing procedure (subgroup 1) and twenty samples were exposed to a toothbrushing simulation process (subgroups 4i and 4ii).

### 2.3. Simulation of Acid Attack

A solution of hydrochloric acid with a concentration of 30% and a pH of 2.12 was used to simulate the acid attack produced by gastric acid. The pH value was established with a portable pH meter (Thermo Scientific Eutech pH 5+, Vernon Hills, IL, USA) The submersion of the samples in acid was carried out in a single cycle of 60 min, in an incubator, at a constant temperature of 37 °C. After this stage, the samples were stored in distilled water at 37 °C. Ten samples from each group were not further submitted to toothbrushing simulation process (subgroup 5).

### 2.4. Brushing Simulation

This stage was performed immediately after acid submersion for twenty samples in each group (subgroups 2i and 2ii) and 30 min after acid submersion for another twenty samples in each group (subgroups 3i and 3ii). A brushing simulation device was used at a frequency of 5000 brushing cycles with an intensity of 100 cycles/minute and a constant load of 200 g. Brushing was performed for a half of the samples in each group using toothbrushes with medium bristle hardness (Toothbrush R.O.C.S. Professional Medium, Tallinn, Estonia) (subgroups 2i, 3i, 4i) and for the other half of the samples using hard bristle hardness (Toothbrush R.O.C.S. Professional Firm, Tallinn, Estonia) (subgroups 2ii, 3ii, 4ii) and a tooth paste slurry obtained by mixing a toothpaste (Sensodyne, GSK, Middlesex, UK) and distilled water in 1:3 ratio. The characteristics of the toothbrushes are presented in Table 2. After performing this step, the samples were rinsed under running water and dried for 2 min using the air spray from the dental unit.

### 2.5. Profilometry

Surface characteristics of all the samples after finishing and polishing procedure, acid challenge and toothbrushing cycles were evaluated using profilometry. The arithmetic deviation of the evaluated profile values (Pa) were recorded using a noncontact profilometer Dektak XT (Bruker, Tuscon, AZ, USA). For each sample, we reported the mean (Pa) as a result of three determinations. Pa is the arithmetic mean deviation of the primary profile (P).

### 2.6. Statistical Analysis

The data were stored in a Microsoft Office Excel document. IBM SPSS 26 software was used for statistical analysis of the values between and within the groups and subgroups. Parametric ANOVA and Bonferroni post hoc tests were used at a *p* < 0,05 significance level.

## 3. Results

**Figure 2:**
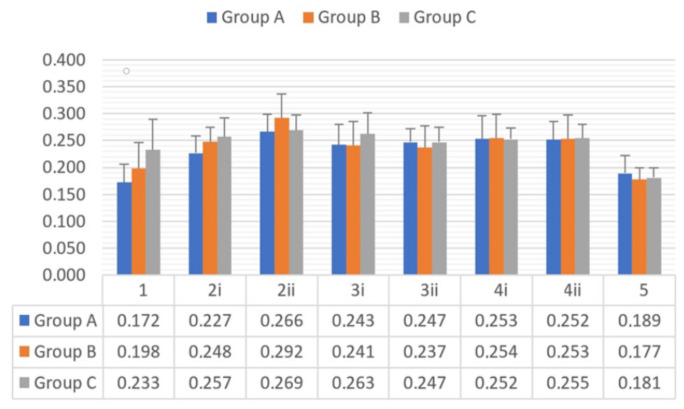
Mean Pa values and standard deviation of each study subgroup by the end of each stage.

**Figure 3:**
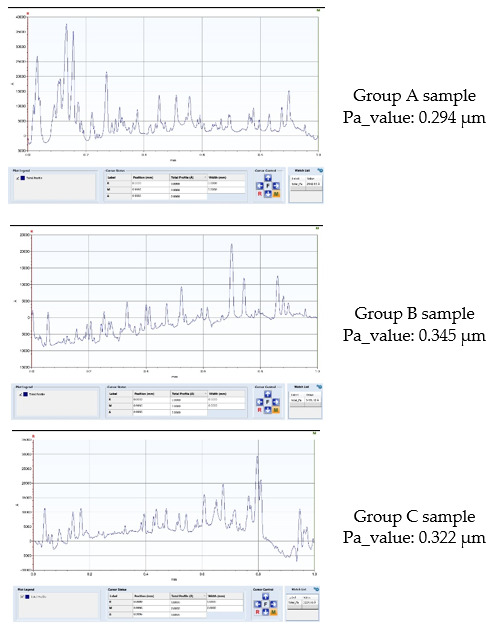
Representative profilometry scan aspect of three samples from groups (**A**–**C**) in subgroup 2ii.

Mean surface roughness values (Pa) and standard deviation in groups and subgroups are presented in Figure 2. Aspects of some profilometric measurements of three samples from groups A, B and C in subgroup 2i are presented in Figure 3. There were no statistically significant differences between groups A, B and C in any of the study subgroups (Table 3).

Within the study groups, significant differences were found between the same subgroups by the end of the stages (Table 4).

No significant differences were found between groups A, B and C when comparing the results in each subgroup (Table 3).

Statistical test results regarding the differences between the subgroups in groups A, B and C are presented in Table 4. In group A, significantly higher Pa values were recorded between subgroup 1 and 2ii (*p* = 0.003 < 0.05), between subgroups 1 and 3ii (*p* = 0.039), subgroups 1 and T4i (*p* = 0.019), subgroups 1 and 4ii (*p* = 0.021) and between subgroups 5 and 2ii (*p* = 0.033). In group B, significantly higher Pa values were recorded between subgroups 1 and 2ii (*p* = 0.024) and subgroups 2ii and 5 (*p* = 0.002). In group C, significantly higher differences were recorded between subgroups 5 and 2i (*p* = 0.031), subgroups 5 and 2ii (*p* = 0.006), subgroups 5 and 3i (*p* = 0.014) and subgroups 5 and 4ii (*p* = 0.04).

## 4. Discussion

The present study investigated the surface roughness of three types of flowable composite resins after exposure to acid attack with hydrochloric acid and after toothbrushing simulation with toothbrushes with bristles of different hardness and toothpaste with a medium abrasive value performed immediately after submersion to acid, 30 min after acid attack or without prior chemical challenge.

The samples were subjected to finishing and polishing procedure using Sof-Lex system, and the results showed that the average surface roughness values were less than 0.2μm for two of the three flowable composite resins used in the study (Grandio Flow and Filtek Ultimate Flow), in accordance with the results of a study conducted by Somacal et al. [5,11]. Yuan et al. noted that bacterial adhesion is also influenced by surface energy, not only by surface roughness [12]. An example of this would be that S mutans have a higher tendency to adhere to substrates with high surface energy, the latter being influenced by the composition of composite resin fillers [5,12,13]. Other studies have shown that composite resins with an organic matrix based on high molecular weight monomers such as Bis-GMA are more resistant and harder to remove by abrasive procedures, exfoliating a smaller number of inorganic particles [14,15].

The wear resistance of composite resins depends on the type of material and is influenced by the particularities of the organic matrix; the filler particles; respectively, the volume and weight in percent; their size, shape and distribution; and the matrix–filler interaction [16]. A number of studies have concluded that flowable composite resins have a higher wear resistance due to the shorter distance between the particles and the presence of small filler particles [16,17]. This idea is also supported by the results of other studies, Condon et al., Sulong et al. and Suzuki et al., which argue that small particles give the matrix greater wear resistance [18,19,20]. Another study concluded that both flowable and higher viscosity resins have a relatively low wear rate, and there are no statistically significant differences between the two types of composite resins [16]. On the other hand, Hashemikamangar argues that flowable composite resins are less resistant to wear compared to higher viscosity resins [21]. In the present study, it was observed that there were no statistically significant differences between the three types of fluid composite resins tested (Grandio Flow, Filtek Ultimate Flow and G-aenial Flo X) at any stage of the study, although the percentage of the weight and volume of filler particles are different for all materials used in the study. In another study, it was reported that there was no correlation between the surface roughness obtained after the finishing and polishing procedures and the final wear of resin-based materials [5,22].

Data from the literature considered that the degradation of composite resins is caused by the chemical degradation of polymers following the penetration of water into its structure and the consequent release of oligomers and monomers through the pores created by mechanical wear [5]. It was found that, initially, there is a superficial degradation of the polymer, and later the surface roughness increases by the appearance of cracks due to increased osmotic pressure at the interface between the organic matrix and filler particles, respectively, due to hydrolytic degradation of silane [23,24,25,26,27].

In the present study, hydrochloric acid determined changes in surface roughness of all three flowable composite resins, the results being in agreement with the results of other studies [24,28].

The methodology for simulating toothbrushing is well established in the literature. Thus, Sexson and Phillips claimed that a patient performs about 15 cycles for each brushing session [29]. Thus, two brushing sessions per day will lead to a total number of 10,000 brushing cycles in a year [5,30,31]. In the present study, we subjected the study samples to a session of 5000 brushing cycles, equivalent to 6 months of toothbrushing. In a study conducted by AlAli et al., it was demonstrated that after a toothbrushing procedure of 5000 cycles, the surface polished with Sof-Lex system had been removed, and the exposed layers were more resistant to the following toothbrushing cycles [14].

In our study, we recorded higher Pa values of surface roughness for the subgroups subjected to the abrasive action of hard bristles. These results are inconsistent with those obtained in the study conducted by Carvahlo et al. in which soft bristles created a rougher surface due to the ability of soft bristles to hold toothpaste better and the flexibility of the filaments, which ensures a larger contact area between toothbrushes or toothpaste and restorative materials [32]. From a clinical point of view, the increase of the surface roughness of restoration materials will reduce wear resistance and increase the accumulation of bacterial biofilm, leading to secondary caries or impaired aesthetics [2,32].

The differences in composition of organic and inorganic content of flowable composite resins influence their behavior in acid attack. In the case of the tested composite resins, there are differences both in terms of organic and inorganic components so that it is difficult to specify what contributed to the similar behavior of the materials.

Gaining a better understanding of how surface roughness is affected by the characteristics of filler particles, the resin matrix and the connection between the matrix and the filler material could help to choose the ideal toothbrushes, toothpastes or the proper moment to perform toothbrushing [5,33].

## 5. Conclusions

Hydrochloric acid action for 60 min and six months of toothbrushing using toothbrushes having medium hardness or firm bristles have no effect on the surface roughness of the tested flowable composite resins. Toothbrushing with firm bristles immediately after acidic challenge determines increased surface roughness for two of the three flowable composite resins (Grandio Flow and Filtek Ultimate Flow). Toothbrushing with medium or firm bristles thirty minutes after the acidic aggression determines no effect on surface condition of flowable composite resins. The results obtained open new perspectives regarding the dental restorative treatment with flowable composite resins in patients with gastroesophageal reflux disease.

## Figures and Tables

**Table 1:** Detailed presentation of the materials used in the study.

The Name of Flowable Composite Resin	Manufacturer	Batch No.	Composition	Filler wt%/vol%
Grandio Flow	VOCO GmbH, Cuxhaven, Germany	2036127	Bis-GMA, TEGDMA, HEDMA, glass ceramic, nanoparticle	65.6 wt%/80 vol%
Filtek Ultimate Flow	3M-ESPE, St. Paul, MN, USA	3930A2	Bis-GMA, UDMA, Bis-EMA, Zirconia/silica, zirconia, silica	78.5 wt%/63.3 vol%
G-aenial Flo X	GC Europe	1910162	UDMA, Bis-MEPP, TEGDMA, silicon, dioxide, strontium glass	69 wt%/50 vol%

Bis-GMA—Bisphenol A diglycidyl ether methacrylate; Bis-MEPP—Bisphenol 4-methacryloxypolyethoxyphenil propane; Bis-EMA—Bisphenol-A ethoxylated dimethacrylate; TEGDMA—Triethylenglycol dimethacrylate; UDMA—Urethane dimethacrylate; HEMA—Hydroxyethyl methacrylate.

**Table 2:** Characteristics of the toothbrushes.

Toothbrush Name	Bristle Hardness	Bristle Material	Bristle Length	Bristle Thickness
R.O.C.S. ”Professional” Medium	medium	nylon	0.8/1.3	0.18/0.2
R.O.C.S. ”Professional” Firm	hard	nylon	Not provided by the producer	Not provided by the producer

**Table 3:** Differences between the study subgroups of each study group.

Subgroups	1			2i			2ii			3i			3ii			4i			4ii			5		
Groups	A	B	C	A	B	C	A	B	C	A	B	C	A	B	C	A	B	C	A	B	C	A	B	C
A	-	*	*	-	*	*	-	*	*	-	*	*	-	*	*	-	*	*	-	*	*	-	*	*
B	*	-	*	*	-	*	*	-	*	*	-	*	*	-	*	*	-	*	*	-	*	*	-	*
C	*	*	-	*	*	-	*	*	-	*	*	-	*	*	-	*	*	-	*	*	-	*	*	-

* Statistically not significant (*p* < 0.05).

**Table 4:** Differences between the study subgroups in groups A, B and C.

	Group A								Group B								Group C							
	1	2i	2ii	3i	3ii	4i	4ii	5	1	2i	2ii	3i	3ii	4i	4ii	5	1	2i	2ii	3i	3ii	4i	4ii	5
1	-	*	**	*	**	**	**	*	-	*	**	*	*	*	*	*	-	*	*	*	*	*	*	*
2i	*	-	*	*	*	*	*	*	*	-	*	*	*	*	*	*	*	-	*	*	*	*	*	**
2ii	**	*	-	*	*	*	*	**	**	*	-	*	*	*	*	**	*	*	-	*	*	*	*	**
3i	*	*	*	-	*	*	*	*	*	*	*	-	*	*	*	*	*	*	*	-	*	*	*	**
3ii	**	*	*	*	-	*	*	*	*	*	*	*	-	*	*	*	*	*	*	*	-	*	*	*
4i	**	*	*	*	*	-	*	*	*	*	*	*	*	-	*	*	*	*	*	*	*	-	*	*
4ii	**	*	*	*	*		-	*	*	*	*	*	*	*	-	*	*	*	*	*	*	*	-	**
5	*	*	**	*	*	*	*	-	*	*	**	*	*	*	*	-	*	**	**	**	*	*	**	-

** Statistically significant (*p* < 0,05); * Not significant.
